# CD24-mediated neutrophil death in inflammation: *ex vivo *study suggesting a potential role in sepsis

**DOI:** 10.1186/cc11768

**Published:** 2012-11-14

**Authors:** M Parlato, F Souza-Fonseca-Guimaraes, F Philippart, B Misset, M Adib-Conquy, JM Cavaillon

**Affiliations:** 1 Institut Pasteur, Paris, France; 2 Saint Joseph Hospital, Paris, France

## Background

Delayed neutrophil apoptosis is often detected in inflammatory pathologies, including sepsis [1]. CD24 is a small heavily glycosylated cell-surface protein, linked to the membrane by a glycosyl-phosphatidylinositol (GPI) anchor in a wide variety of cells [2]. Cross-ligation of CD24 triggers apoptosis in a human B-cell subset during the early activation stage [3]. Since CD24 is also expressed on neutrophils, we aimed to study its expression in sepsis and its putative role in apoptosis.

## Methods

Blood samples were either collected from healthy donors or from sepsis patients at the onset of sepsis and the two following days and surface CD24 expression on neutrophils was analyzed by flow cytometry. Peripheral blood neutrophils were purified from sepsis patients and healthy individuals by positive selection. Whole blood or neutrophils were challenged with lipopoplysaccharide (LPS), TNF, granulocyte-macrophage colony-stimulating factor (GM-CSF), heat-killed *Staphylococcus aureus *or heat-killed *Escherichia coli *and CD24 expression assessed by flow cytometry. Neutrophils were cross-linked with anti-human CD24 and assessed for apoptosis after 24 hours of culture. In some experiments, neutrophils were preincubated with caspase inhibitor (z-VAD-fmk) before crosslinking.

## Results

Surface expression of CD24 assessed by flow cytometry was significantly altered in neutrophils from sepsis patients compared with healthy controls. Activation of neutrophils with LPS or heat-killed bacteria in whole blood triggers a strong upregulation of CD24 at surface level despite no enhanced expression being observed when the activation was carried out in purified neutrophils. In contrast, TNF and GM-CSG upregulated CD24 in whole blood and in purified neutrophils. CD24 cross-ligation induces caspase-independent apoptosis in human neutrophils. *Ex vivo *death responses after CD24 ligation in neutrophils from sepsis patients are currently under study.

## Conclusion

This is the first report studying the role of CD24 in sepsis patients, linking homeostasis and apoptosis.
